# A panel of microsatellites to individually identify leopards and its application to leopard monitoring in human dominated landscapes

**DOI:** 10.1186/1471-2156-10-79

**Published:** 2009-12-04

**Authors:** Samrat Mondol, Navya R, Vidya Athreya, Kartik Sunagar, Velu Mani Selvaraj, Uma Ramakrishnan

**Affiliations:** 1 National Centre for Biological Sciences, TIFR, GKVK Campus, Bellary Road, Bangalore 560065, India; 2 Kaati Trust, D3, Raanwara, Bavdhan, Pune 411021, India

## Abstract

**Background:**

Leopards are the most widely distributed of the large cats, ranging from Africa to the Russian Far East. Because of habitat fragmentation, high human population densities and the inherent adaptability of this species, they now occupy landscapes close to human settlements. As a result, they are the most common species involved in human wildlife conflict in India, necessitating their monitoring. However, their elusive nature makes such monitoring difficult. Recent advances in DNA methods along with non-invasive sampling techniques can be used to monitor populations and individuals across large landscapes including human dominated ones. In this paper, we describe a DNA-based method for leopard individual identification where we used fecal DNA samples to obtain genetic material. Further, we apply our methods to non-invasive samples collected in a human-dominated landscape to estimate the minimum number of leopards in this human-leopard conflict area in Western India.

**Results:**

In this study, 25 of the 29 tested cross-specific microsatellite markers showed positive amplification in 37 wild-caught leopards. These loci revealed varied levels of polymorphism (four-12 alleles) and heterozygosity (0.05-0.79). Combining data on amplification success (including non-invasive samples) and locus specific polymorphisms, we showed that eight loci provide a sibling probability of identity of 0.0005, suggesting that this panel can be used to discriminate individuals in the wild. When this microsatellite panel was applied to fecal samples collected from a human-dominated landscape, we identified 7 individuals, with a sibling probability of identity of 0.001. Amplification success of field collected scats was up to 72%, and genotype error ranged from 0-7.4%.

**Conclusion:**

Our results demonstrated that the selected panel of eight microsatellite loci can conclusively identify leopards from various kinds of biological samples. Our methods can be used to monitor leopards over small and large landscapes to assess population trends, as well as could be tested for population assignment in forensic applications.

## Background

The cat family *Felidae *(order *Carnivora*) originated in the late Miocene [1] and has evolved into one of the world's most successful carnivores. The leopard, *Panthera pardus *is the most widely distributed and adaptable large cat in this family. The geographical range of leopards spanned all of sub-Saharan and North Africa, the Middle East and Asia Minor, South and Southeast Asia, and extended to the Amur Valley in the Russian Far East [2,3]. With nine extant genetic subspecies [4,5] reduced from earlier 27 morphospecies [6,7], leopards still inhabit a significant portion of their historic range, although their numbers have been significantly reduced due to massive habitat loss and anthropogenic impacts [5].

Unlike other species of large cats, leopards in India commonly occur outside protected areas where the potential for conflict is high and where logistics, expenses, ethical issues make detailed ecological studies on this secretive species using standard field methods [8] difficult. Non-invasive genetic typing of DNA extracted from animal hair or scats have been used for individual identification of rare, endangered or cryptic species [9-16]. Such methods would be ideal for monitoring leopards in human-dominated landscapes.

Unambiguous 'genetic individual identification' requires data from multiple loci [17,18] depending on the population size of any species in any given area [19]. Studies on Arabian leopards (*Panthera pardus nimr*) used non-invasive samples for estimating populations [15]. However, these methods were developed in the context of surveying relatively small populations. In this paper, we have developed a set of methodological tools for the genetic individual identification of leopards aimed at larger landscapes, where higher numbers of leopards are likely to be present (Athreya *et al. *unpublished). Specifically, our objectives were (1) to identify a set of hypervariable microsatellite loci for the individual identification of leopards and (2) to test the feasibility of using these loci for leopard individual identification from non-invasive samples (feces) collected from a human-dominated landscape.

## Results

### Standardization of Individual identification

#### Microsatellite amplification

We initially tested a total of 29 microsatellites using leopard blood DNA to determine the most polymorphic loci. 25 loci (out of 29) showed amplification (Table 1) while four did not (FCA069, FCA311, FCA466 and F27). The microsatellite loci varied from highly polymorphic (locus FCA506-12 alleles; loci FCA628 and FCA391, *H*_*e *_= 0.79) to less polymorphic (loci FCA170, FCA164, FCA453 and E21B- four alleles; loci FCA170 and FCA164, *H*_*e *_= 0.05). Only one of the 25 loci (locus F115) was significantly out of Hardy-Weinberg equilibrium (see Table 1). We did not find evidence for linkage disequilibrium between any pair of loci. We selected an initial screening set of 10 loci (Table 1) for individual identification due to their short amplicon size (which should result in high amplification success from potentially degraded fecal DNA samples) and high cumulative P_ID(sibs) _value.

**Table 1:** Genetic variability at 25 microsatellites for 37 leopards (blood samples)

Locus	Repeat nature	Dye	Product size range (bp)	T_a_	No. of alleles	H_e_	H_o_	P_ID(unrelated) _cumulative	P_ID(sibs) _cumulative	Amplification success (%) for faeces	Allelic dropout (%)	Multiplex sets for PCR
FCA126*^$^	Di	6-FAM	134 - 154	57.0	10	0.85	0.74	3.975E-02	3.356E-01	94%	0	Set 1
FCA279*^$^	Di	NED	81 - 105	57.0	11	0.83	0.68	1.882E-03	1.156E-01	88%	1.5	
FCA304*^$^	Di	NED	105 - 129	58.0	8	0.80	0.37	1.255E-04	4.229E-02	88%	0	Single reaction
FCA052*^$^	Di	6-FAM	107 - 121	55.0	8	0.74	0.50	8.721E-08	2.523E-03	92.1%	1.5	Set 2
FCA090*^$^	Di	HEX	104 - 116	55.0	6	0.74	0.71	8.819E-10	4.058E-04	77%	3	
FCA672*^$^	Di	6-FAM	82 - 100	54.0	8	0.74	0.68	8.564E-12	6.655E-05	92.1%	0	Single reaction
FCA230*^$^	Di	HEX	98 - 106	52.0	5	0.70	0.71	1.572E-15	2.098E-06	90%	6	Single reaction
FCA309*^$^	Di	6-FAM	92 - 106	55.0	6	0.67	0.58	8.754E-19	8.788E-08	94%	0	Single reaction
FCA205*	Di	NED	89 - 113	55.5	9	0.76	0.71	1.108E-05	1.646E-02	22%	3	
FCA628*	Di	HEX	98 - 110	58.0	7	0.73	0.79	8.045E-11	1.638E-04	48%	9	
F42	Tetra	NED	226 - 250	55.4	7	0.77	0.68	9.204E-07	6.404E-03			
FCA391	Tetra	HEX	190 - 218	56.0	8	0.76	0.79	8.920E-09	1.006E-03			
6HDZ056	Di	HEX	169 - 177	58.0	5	0.63	0.53	9.242E-13	2.709E-05			
F115^†^	Tetra	HEX	190 - 206	55.5	5	0.75	0.32	9.552E-14	1.106E-05			
FCA506	Di	6-FAM	192 - 226	54.0	12	0.72	0.68	1.285E-14	4.786E-06			
F41	Tetra	NED	123 - 139	56.0	5	0.68	0.68	2.381E-16	9.303E-07			
6HDZ170	Di	6-FAM	212 - 226	60.0	6	0.69	0.61	3.581E-17	4.152E-07			
FCA001	Di	6-FAM	139 - 163	54.0	10	0.68	0.66	5.968E-18	1.897E-07			
FCA453	Tetra	6-FAM	181-201	59.0	4	0.64	0.55	1.643E-19	4.181E-08			
6HDZ007	Di	HEX	202 - 226	65.0	10	0.65	0.50	3.022E-20	2.018E-08			
E21B	Di	6-FAM	157 - 167	60.0	4	0.58	0.58	7.733E-21	1.063E-08			
FCA232	Di	6-FAM	113 - 123	55.0	5	0.55	0.71	2.046E-21	5.749E-09			
FCA441	Tetra	HEX	137 - 153	50.0	5	0.53	0.50	5.034E-22	3.129E-09			
FCA164	Di	6-FAM	80 - 90	58.0	4	0.10	0.05	4.073E-22	2.821E-09			
FCA170	Di	HEX	84 - 94	55.0	4	0.10	0.05	3.296E-22	2.544E-09			

* Loci tested for faecal DNA samples

^$ ^Loci used for individual identification

^† ^Deviation from Hardy-Weinberg equilibrium

We tested these 10 loci with 18 fecal samples from captive individuals, for which we had the corresponding blood DNA samples. Two of these samples showed poor amplification (for three and one locus respectively), and were removed from subsequent analyses.

Overall, amplification success ranged from 22-94% for the fecal DNA samples from captive individuals. We dropped the two loci with amplification success lower than 77% (FCA205 (22% success) and FCA628 (48% success), Table 1) from further analyses. Eight (of ten) loci revealed an amplification success ≥77% (Table 1). These eight loci were multiplexed into six PCR reactions for different sources of DNA (Table 1).

#### Individual identification and data validation

Of the 18 fecal samples collected from captive wild caught individuals, only 16 produced five or more loci with quality index ≥0.75. Eight of these samples produced data for all the eight loci. We could generate genetic data for seven and six loci genotype profiles from five and three samples, respectively. The estimated cumulative probability of identity assuming all individuals are siblings (P_ID(sibs)_) was 2 × 10^-9^, based on 25 microsatellite loci. The selected eight loci for non-invasive DNA samples resulted in a P_ID(sibs) _of 5 × 10^-4 ^(1 in every 2000 leopards). For all 10 loci, the allelic dropout rate ranged from 0 - 9%. Four of the loci showed no allelic dropout, while the remaining six loci revealed dropout rates of 1.5-9% from seven samples. The multilocus genotypes from the fecal samples exactly matched the blood genotypes from the same individuals.

#### Individual identification of leopards in a human-dominated landscape

##### Species identification

The leopard-specific primer produced a strong band of around 130 bp from known leopard DNA samples. Sequencing of these bands confirmed the amplicons to be leopard sequences. No positive amplifications were observed from any other tested carnivore or potential prey species (see methods). We did not observe any non-specific amplification that could be attributed to nuclear inserts.

Out of 141 fecal samples collected from the study area, genetic screening confirmed leopard origin from 44 samples (31.2%). The remaining samples did not show any amplification with the leopard-specific primers.

##### Microsatellite amplification from leopards

We amplified the set of eight loci from all the 44 field leopard fecal samples. Only 13 samples (29%) produced data for six or more loci data during the initial screening. Ten of these samples (~23%) produced reliable data after all the trials described in the methods, and were used for further analyses. Amplification success ranged from 46-72% (Table 2).

**Table 2:** Genetic variability at 8 microsatellites for 7 field-collected leopards (fecal samples)

Locus	No. of alleles	H_e_	H_o_	P_ID(unrelated) _cumulative	P_ID(sibs) _cumulative	Amplification success (%) for faeces	Allelic dropout (%)	False alleles (%)
FCA126	6	0.77	0.67	1.695e-02	3.886e-01	48	6.45	0
FCA090	5	0.73	0.67	9.579e-04	1.599e-01	52.38	0	0
FCA052	4	0.69	0.44	7.947e-05	7.125e-02	54.83	7.40	1.5
FCA672	4	0.69	0.67	6.593e-06	3.174e-02	64.22	2.22	0
FCA279	4	0.68	0.67	6.004e-07	1.428e-02	46.09	0	0
FCA304^†^	5	0.66	0.22	6.677e-08	6.665e-03	47.5	6.66	0
FCA230	4	0.63	0.44	8.167e-09	3.219e-03	46.77	0	0
FCA309	3	0.62	0.78	1.287e-09	1.598e-03	72.41	2.63	1.47

^† ^Deviation from Hardy-Weinberg equilibrium

Nine of the ten field collected samples produced data for all the eight loci with the remaining producing result for seven loci. We could identify seven different individuals based on six loci (see [16] for details), with a P_ID(sibs) _value of 1 × 10^-3^. Locus FCA304 was found to be significantly out of Hardy-Weinberg equilibrium (see Table 2). Allelic dropout rate ranged from 2.2 - 7.4% and two of the loci (FCA052 and FCA309) produced false alleles (Table 2). Six of the samples showed allelic dropout for 1 or 2 loci.

## Discussion

In this paper, we standardized genetic individual identification of leopards from different sources of DNA with protocols offering some advantages over past efforts [15]. Firstly, species identification of the field-collected samples ensured that only leopard fecal samples were used for further analysis. Initial screening of samples (for species identification) is critical in landscapes where multiple, similar-sized carnivore species co-occur. In addition, use of a sufficiently large panel of 25 microsatellite loci for the initial assessment of variability improved our ability to ascertain the combination of loci resulting in higher precision in probability of identity. This enabled us to get an optimal combination of a few very variable loci. Our results revealed that eight loci provide an adequate probability of identity rate for population estimation in larger landscapes (with a large leopard population size). Also, this approach allowed us to exclude loci that might be problematic for non-invasive samples. For example, loci FCA205 and FCA628 showed high level of polymorphism with the blood DNA samples, but very low amplification success (22% and 48% respectively) and high genotyping error rate (3% and 9% respectively), resulting in removal of these markers from the initial set. Overall, our results are in accordance with the review by Broquet *et al. *[20], with higher amplification success for dinucleotide loci.

Perez *et al. *[15] performed individual identification with four microsatellite loci in Arabian leopards. Although they tested 31 microsatellite markers, they found only four to be polymorphic, which resulted in a P_ID(sibs) _value of 0.56-0.81 [15]. Since they surveyed an area where the population size was around 10 individuals these loci were adequate for their study. However, this set of microsatellites will not provide enough discriminating power to ascertain individuals for population estimation in potentially high leopard density areas, for e.g. Indian subcontinent or Africa. For instance, our analyses produced a P_ID(sibs)_value of 0.03 (for the field samples) with a comparable number of four loci, suggesting that the use of highly polymorphic loci can result in less error-prone individual identification. Our motivation in this study was to develop protocols that could be applied over larger leopard habitats with large leopard populations. Waits *et al. *have recommended that the threshold value of P_ID(sibs) _to sufficiently differentiate among individuals for population estimation should be at least double than the approximate number of animals in any given area [19]. We suggest modifying the number of loci according to the required level of precision and study area/approximate number of individuals for which we seek population estimates.

Fecal samples from field conditions could potentially result in highly variable DNA quality and quantity, resulting in possible errors in genotypes. Our results revealed that field-collected samples had lower amplification success and higher genotyping error rate compared to fecal DNA that was freshly collected from captive individuals. Only 23% of the leopard samples from the field produced enough data for individual identification. This could be because scats from captive individuals were relatively fresh potentially yielding higher quality and quantity of DNA, while those from the field were old and degraded. We also observed fairly low amplification success for two loci with a small amplicon size for the field samples, contrary to expectations from Broquet *et al. *[20].

The initial panel of 25 microsatellites revealed a low level of genetic diversity (average observed heterozygosity = 0.57, s.d. = 0.19) for our samples, compared to a previous study on Indian leopards (Mean observed heterozygosity = 0.69, s.d. = 0.14 from nine samples and 25 loci [5]). The field samples revealed moderate level of genetic diversity (Mean observed heterozygosity = 0.66, s.d. = 0.2). The lower genetic diversity in our samples could be because we included potentially related individuals from a small geographical area.

We applied our panel of microsatellites to non-invasive samples collected from a human-dominated agricultural landscape in the Indian subcontinent. Our results revealed a minimum of seven individuals over the 250 sq km area. Although we did not aim to conduct population estimation in this study, our results indicate that a large number of leopards inhabit a completely human-dominated, agricultural landscape with a population density of > 150/sq. km. Most conservation efforts in India are focused to within protected areas, which constitute only 5% of the countries landmass. Many species of carnivores, like the leopards sampled in this study, occur outside such areas and among high densities of humans. The potential for conflict with humans is also highest in these shared spaces, with attacks on humans as well as livestock making it even more important to obtain ecological information for the conservation of the species as well as for the welfare of the people. In this study we have developed methods that will allow us to estimate leopard populations in larger landscapes outside of protected areas. Apart from their application for population estimation, our set of microsatellite loci can be used for future research to investigate genetic diversity, relatedness, population structure and wildlife forensics for this widely distributed and elusive large cat which is also under severe threat due to illegal poaching.

## Conclusion

We used 25 microsatellite loci to ascertain an optimal set of eight loci that can reliably identify individual leopards from non-invasive DNA samples. Our results revealed a probability of identity of one in 2000 leopards for scats from wild caught individuals. Application of our methods to field collected scats revealed a minimum of seven individuals in a human dominated, agricultural landscape. In combination with a good sampling strategy, our methods can be used in a cost-effective way to investigate species biology (including patterns of genetic diversity, relatedness and population connectivity) as well as to estimate population abundance for leopards in the wild.

## Methods

### Sample collection

Blood samples were collected from 36 wild-caught leopards from Maharastra and one leopard from Himachal Pradesh, India. The leopards were trapped following conflict incidents from six different districts in Maharastra (Mumbai, Thane, Nashik, Ahmednagar, Junnar and Vaijapur). Genetic material was collected from anaesthesized animals. Feces were collected from 18 of these leopards (17 from Maharastra and one from Himachal Pradesh) to test the effects of different sources of DNA (i.e. blood or feces) on the amplification process and to estimate the associated error. All scat samples were carefully collected to avoid contamination in ethanol as recommended by Murphy *et al. *[21] and stored in room temperature till DNA extraction, which was performed after more than six months.

The field samples were collected from a human-dominated agricultural landscape where people's density is about 200/sq km. Since this was the first time such an area was being sampled, we decided to sample across all different sub-habitats (mud paths, bunds of fields, tar roads, near houses, dry stream beds etc.) throughout the study area which spanned 250 sq km. We used Google Earth http://earth.google.com to mark a total of 130 km of pathways in the landscape. These paths were surveyed thrice between December 2007 and April 2008 with a three weeks interval between each sampling occasion. We collected all feces on the paths which had remains of hair/poultry and stored them in sterile vials containing alcohol for future DNA analysis. A total of 141 fecal samples were collected during the survey and all collections were carried out with appropriate permits from the Government of India.

### DNA Extraction

DNA was extracted from blood using QIAamp DNA Tissue Kit (QIAGEN Inc) following the manufacturer's instructions. The DNA extraction was performed following protocols described in Mondol *et al. *[16]. DNA was eluted in 100 μl of TE buffer (ph 7.8) and conducted in a separate, pre-PCR laboratory space under sterile conditions to minimize contamination.

### Species identification

In this study, we developed a novel PCR based species identification for leopards to eliminate misidentified fecal samples from other species with similar body sizes. We designed leopard-specific mitochondrial DNA primers from NADH4 region using similar approach described in Mukherjee et al. [22]. The primer sequences are forward: TRATAGCTGCYTGATGAC and reverse: GTTTGTGCCTATAAGGAC.

### Microsatellite primer selection

We selected 29 micosatellites designed from domestic cat [23] and different tiger subspecies including *Panthera tigris sumatrae *[24] and *Panthera tigris tigris *[14]. The primers were selected based on the number of alleles and the level of observed heterozygosity (H_obs_) in these species. Of the 29 loci, 23 had dinucleotide motifs and six had tetranucleotide motifs.

### PCR standardization and genotyping

All PCR standardizations were initially conducted using the blood and fecal DNA samples from wild caught captive leopards. In addition, we also tested the leopard specific primers for cross-amplification from other carnivores (e.g., tiger (*Panthera tigris tigris*), wild dog (*Cuon alpinus*), domestic dog (*Canis familiaris*), jackal (*Canis aureus*), striped hyena (*Hyena hyena*), sloth bear (*Melursus ursinus*), jungle cat (*Felis chaus*), domestic cat (*Felis catus*)) as well as potential herbivore prey species like sambar (*Cervus unicolor*), spotted deer (*Axis axis*) and gaur (*Bos gaurus*). The PCR conditions included an initial denaturation (95°C for 15 min); 50 cycles of denaturation (94°C for 30 secs), annealing (50°C for 30 secs) and extension (72°C for 30 secs); followed by a final extension (72°C for 10 min). The PCR products were visualized in a 2% agarose gel and then cleaned by Exo-Sap mixture (NEB) and sequenced from both ends on an ABI 3100XL capillary sequencer. All sequences were visualized and edited manually.

For all the microsatellite primer standardizations, amplification was carried out in 10 μl reaction volumes containing 5 μl Qiagen multiplex PCR buffer mix (QIAGEN Inc.), 0.2 μM labeled forward primer (Applied Biosystems),0.2 μM unlabelled reverse primer, 4 μM BSA and 1 μl of the DNA extract. For fecal DNA, 3 μl of the extract was used in the reaction mixture. The temperature regime included an initial denaturation (94°C for 15 min); 30 cycles of denaturation (94°C for 30 secs), annealing (T_a _for 45 secs) and extension (72°C for 45 secs); followed by a final extension (72°C for 30 min) in an Eppendorf thermocycler. PCR negatives were incorporated in all reaction setups to monitor contamination. The optimum number of PCR cycles was standardized to be 30 and 40 cycles for blood and scat DNA samples, respectively. The PCR product was visualized in a 2% agarose gel. 1 μl of the amplified product was added into 10 μl of formamide and 0.5 μl of ROX 500 size standard and then run into an automated sequencer ABI3100XL (Applied Biosystems). Microsatellite alleles were scored with GENEMAPPER version 4.0 (Applied Biosystems).

We amplified microsatellites from different DNA extracts to compare the DNA quality from both the extracts. Average amplification success for each of the tested loci from the fecal samples was calculated as the percent positive PCR as described by Broquet *et al. *[20]. We quantified the average allelic dropout (across individuals) and the average number of false alleles manually as the number of dropouts or the number of false alleles over the total number of amplifications respectively as described by Broquet and Petit [25]. Further, we compared the results from blood and fecal DNA genotypes and calculated the P_ID(obs) _and the P_ID(sibs) _for the blood samples using GIMLET [26]. Finally, 10 dinucleotide microsatellite loci were selected for further analyses based on their cumulative P_ID(sibs) _value, size and repeat motifs and their ability to be multiplexed with other loci. These 10 microsatellite loci were combined into six PCR reactions (information provided in Table 1).

### Data Validation

To counteract the varying quality multilocus genotypes produced from relatively bad DNA sources (in this case, feces) we have followed a modified multiple tube approach combined with a quality index approval as explained by Mondol *et al. *[16]. For the field-collected samples we performed a selection process for individual identification: (i) all samples were amplified and genotyped twice at all eight loci, those samples for which allele sizes could be determined for at least six loci were used for further analysis, while the rest were discarded; (ii) samples which met the above criteria were amplified two more times at all eight loci. Samples producing identical genotypes for at least three independent amplifications for each of the loci were considered reliable. All uncertain genotypes and unamplified samples were further amplified twice. Finally, samples with less than six loci reliable data after all trials were discarded.

### Individual identification

All the 18 fecal DNA samples from captive individuals were genotyped for the ten microsatellite loci. After using a quality index to filter out the low quality samples, the remaining samples were analyzed using the identity analysis module in program CERVUS [27] to identify samples with identical genotypes. For the field collected samples we performed individual identification analysis as described in Mondol *et. al. *[16]. Tests for deviations from Hardy-Weinberg equilibrium were performed for each locus using GENEPOP web version of 3.4 http://genepop.curtin.edu.au/index.html. ARLEQUIN [28] was used to test linkage disequilibrium between loci.

## Authors' contributions

SM designed and executed the laboratory experiments, conducted analyses and wrote the manuscript. NR performed experiments on the field-collected samples, conducted analyses and helped write the manuscript. VA designed the collection protocol, collected the samples and edited the manuscript. VMS and KS conducted experiments and conducted preliminary analyses. UR contributed funding and laboratory space, supervised experiments and analyses and participated in writing the manuscript. All the authors read and approved the final manuscript.
